# DNA Damage in Human Amniotic Cells: Antigenotoxic Potential of Curcumin and *α*-Lipoic Acid

**DOI:** 10.3390/antiox10071137

**Published:** 2021-07-17

**Authors:** Filomena Mottola, Marianna Santonastaso, Concetta Iovine, Cristina Rossetti, Valentina Ronga, Lucia Rocco

**Affiliations:** 1 Department of Environmental, Biological and Pharmaceutical Sciences and Technologies, University of Campania Luigi Vanvitelli, 81100 Caserta, Italy; filomena.mottola@unicampania.it (F.M.); concetta.iovine@unicampania.it (C.I.); 2 Department of Woman, Child and General and Special Surgery, University of Campania, Luigi Vanvitelli, 80138 Napoli, Italy; marianna.santonastaso@unicampania.it; 3 Laboratory of Animal Cytogenetics and Genomics, National Research Council (CNR), ISPAAM, 80055 Napoli, Italy; cristina.rossetti@cnr.it; 4 Prenatal Diagnosis Unit, Varelli Diagnostic Institute, 80126 Napoli, Italy; valentina.ronga@istitutovarelli.it

**Keywords:** human amniocytes, DNA damage, antioxidants, antigenotoxicity, oxidative stress, α-lipoic acid, curcumin

## Abstract

Oxidative imbalances in the gestational phase are responsible for certain complications during pregnancy and for foetal and neonatal genetic disorders. In this work, using human amniocytes, we aimed to evaluate the protection provided to foetal DNA by two concentrations of antioxidant molecules, α-lipoic acid (LA) and curcumin (Cur), against hydrogen peroxide (H_2_O_2_)-induced damage. Genotoxicity tests, performed by the random amplification of polymorphic DNA (RAPD-PCR) technique and TUNEL tests, showed that the lowest concentration of LA-protected cells and DNA from H_2_O_2_ insults. However, a greater ability to protect the amniocytes’ DNA against H_2_O_2_ was observed following co-treatment with the highest concentration of Cur with H_2_O_2_. In fact, a genomic template stability (GTS%) similar to that of the negative control and a statistically significant reduction in the DNA fragmentation index (DFI) were revealed. Moreover, following a combined treatment with both antioxidants and H_2_O_2_, no statistical difference from controls was observed, in terms of both induced mutations and DNA breaks. Furthermore, no effect on morphology or cell viability was observed. The results demonstrate the ability of LA and Cur to protect the genetic material of amniocytes against genotoxic insults, suggesting their beneficial effects in pathologies related to oxidative stress.

## 1. Introduction

Reactive oxygen species (ROS) cause damage to various biological molecules, especially DNA. The excessive production of ROS due to external stimuli or following an endogenous process is often responsible for genotoxic damage [1].

ROS, including superoxide (O_2_^•−^) hydrogen peroxide (H_2_O_2_) and the hydroxyl radical (^•^OH), are responsible for the formation of DNA–DNA intermolecular bonds and for modifications of nitrogenous bases, involved in the establishment and transmission of defective molecules and genetic mutations to subsequent generations [2].

This explains the innumerable pathological implications of oxidative stress (OS), which can arise especially in the perinatal stage, as it is a critical phase very susceptible to external perturbations and characterised by minimal foetal antioxidant capacity.

Normal pregnancy is characterised by mild OS: mitochondrial ROS production at low levels in uteroplacental cells is necessary to regulate a variety of placental processes. Placental ROS signalling is predominantly conferred by H_2_O_2_, which plays a regulatory role through the oxidative modification of cysteine residues on target proteins such as enzymes and transcription factors. On the other hand, excess mitochondrial ROS causes uteroplacental dysfunction by damaging cellular macromolecules, which underlies the pathogenesis of preeclampsia and foetal/intrauterine growth restriction (FGR/IUGR) associated with gestational hypoxia. Gestational hypoxia has a major effect on the mitochondria of uteroplacental cells, causing them to overproduce ROS, leading to OS [3,4].

Oxidative imbalances in the gestational phase are responsible not only for certain complications that occur during pregnancy, but also for a series of foetal and neonatal genetic disorders [4]. An excess of ROS, in the prenatal stage, is responsible for the restriction of foetal growth, early labour, iron-deficiency anaemia and in the most severe cases, congenital malformations, abortions or chromosomal abnormalities [5].

The best-known complication related to OS is preeclampsia: a clinical condition in a pregnant woman that, in the worst cases, can hinder normal blood flow to the foetus and, therefore, nutritional supply, resulting in a delay in foetal growth and, sometimes, even intrauterine death. The study conducted by Hung and collaborators (2006) attributed the origin of this syndrome to excessive placental ROS production following ischemia–reperfusion damage [6].

Supporting the involvement of ROS as a preeclampsia risk factor, literature data show that antioxidant activity was reduced in women with preeclampsia; specifically, the coenzyme Q10 was present in very low amounts, suggesting a useful role of this antioxidant in inhibiting the oxidative syndrome [4].

An inadequate intake of micronutrients and essential vitamins is closely related to foetal mortality. Therefore, inefficient global antioxidant activity and a drop in the levels of enzymatic cofactors, such as zinc, copper and manganese, can compromise the outcomes of pregnancy, as well as increase the risk of diseases in adulthood, including cardiovascular disease and type 2 diabetes mellitus [7]. It follows that strengthening the maternal antioxidant defences should improve the outcome of pregnancy, so safe antioxidant molecules, harmless to the mother and foetus, could be used as therapy to counteract and prevent pathological oxidative stress disorders in pregnancy. Attempts to use vitamins C and E or coenzyme Q10 for preventive or therapeutic purposes during pregnancy have largely been abandoned due to their serious negative consequences. In fact, women supplemented with vitamins C and E were at increased risk of developing gestational hypertension and premature rupture of membranes, but they had a decreased risk of abruptio placentae [8].

α-lipoic acid (LA) is a powerful biological antioxidant and a natural cofactor capable of regenerating essential antioxidant molecules, such as coenzyme Q10 and vitamins C and E, as well as repairing proteins, lipids and DNA damaged by OS. Supplementation with LA during pregnancy could be beneficial, as it can protect against diabetic embryopathy and foetal loss and restore diabetic placentas [9]. Moreover, it has been shown to have a positive effect on the treatment of postpartum perineal pain in association with omega-3, allowing a reduction in the intake of analgesic drugs, which are risky during breastfeeding [10].

Curcumin (Cur) is the main constituent of turmeric (*Curcuma longa*). This particular antioxidant molecule is known as a substance generally recognised as safe for which non-mutagenicity and reproductive non-genotoxicity have been demonstrated [11]. In a mouse model study, Cur improved gestational diabetes mellitus (GDM)-related complications by significantly reducing blood glucose, insulin and total oxidative stress. Furthermore, curcumin also had positive effects on the offspring of mothers with GDM, restoring litter size, weight and congenital birth defects, including neural tube defects (NTDs) [12]. Cur also inhibits the activation of placental macrophages in preeclampsia-like mice, indicating that it has anti-inflammatory properties and suggesting that it may be useful in preventing inflammatory complications in pregnancy [13].

Considering the role of antioxidant molecules in restoring oxidative balance, it is necessary to detect their real protective and preventive action against DNA damage during the gestational phase. In this regard, amniotic fluid could represent a valid aid for the in vitro study of foetal alterations, as, containing differentiated, progenitor and stem cells that represent foetal tissues [14], it provides information about any damage to the foetal DNA, as well as being potentially capable of revealing the protection provided by an antigenotoxic agent.

The focus of this work is determining the potential antigenotoxic effects of two concentrations of LA (200 and 300 µM) and Cur (20 and 40 µM) in counteracting H_2_O_2_ (10 mM) genotoxicity with 48, 72 and 96 h of exposure (h) using amniotic cells as an in vitro experimental model. Furthermore, considering that the combination of multiple antioxidants is known to enhance their individual activity, we proposed evaluating the synergistic effects of the combination of the two antioxidant molecules for the same exposure times (48, 72 and 96 h).

The study was conducted by evaluating cellular morphology and viability, the degree of protection against DNA fragmentation, protection from induced mutations and increased genomic stability by means of TUNEL tests, the random amplification of polymorphic DNA (RAPD)–PCR technique and genomic template stability (GTS) calculations.

## 2. Materials and Methods

### 2.1. Chemicals

α-lipoic acid and curcumin were supplied by Merk KGaA (Darmstadt, Germany CAS: 1077-28-7; CAS: 458-37-7). All the substances tested were dissolved in 0.5% absolute ethanol (Thermo Fisher Scientific Waltham, MA, USA, CAS: 64-17-5).

### 2.2. Cell Culture and Exposure Procedure

Amniotic cells were collected from human amniotic fluid of pregnant women undergoing prenatal diagnosis at Varelli Diagnostic Institute (Napoli, Italy). Briefly, amniotic fluid samples were centrifuged at 1500 revolutions per minute (rpm) for 10 min (min) and suspended in Amniomed^®^ Plus, EuroClone medium at 37 °C, a pH of 7.2–7.4 and 5% CO_2_ [15]. After performing prenatal diagnosis, 30 subcultures were pooled and centrifuged at 1500 rpm for 10 min; then, the pellet was suspended in medium and placed onto a plastic culture flask (surface of 25 cm^2^) for culture to cellular confluence. Then, the cells were trypsinized with 1 mL of 1× trypsin-EDTA (Microgem Cat. L0930-100) and divided into eight experimental groups: one culture system was treated with 200 µM LA; one with 300 µM LA; one with 20 µM Cur; one with 40 µM Cur; one was co-treated with 200 µM LA plus 10 mM H_2_O_2_; one was co-treated with 300 µM LA plus 10 mM H_2_O_2_; one was co-treated with 20 µM Cur plus 10 mM H_2_O_2_; one was co-treated with 40 µM Cur plus 10 mM H_2_O_2_. As a negative control, 0.5% ethanol was used, while as a positive control, 10 mM H_2_O_2_ was applied according to our previous results [15].

We also co-tested LA+ Cur alone and in combination with 10 mM H_2_O_2_ to assess the synergistic effect of the two antioxidants in counteracting H_2_O_2_-induced genotoxicity in amniotic cells. The concentrations were chosen on the basis of preliminary tests and literature data [16]. Although other groups have demonstrated the antioxidant and antigenotoxic effects of LA at lower concentration [17], a final LA concentration of 300 μM was selected according to the maximal protective effective dose as determined in vitro by Maddux et al. 2001 [18]. The substances were added as soon as each culture system had reached 80% cell confluence. The exposure times were 48, 72 and 96 h, in order to monitor the full growth cycles of the amniocytes in vitro, which take 48 h to achieve 80% confluence; we tested until 96 h to allow maximum cell confluence to be reached [19].

Between the two concentrations of LA and Cur, one was chosen for co-exposure based on the results obtained from the individual exposures. The same cells pool was used across all assays and experiments. All the experiments were performed in triplicate.

### 2.3. Cell Morphology Analysis

The alterations of the conventional fibroblastoid morphology of amniocytes [16] were investigated by observing cell morphology in terms of sizes and shapes of the treated amniocytes compared to those of the controls. Morphological observations were performed using an inverted optical microscope (Optika XDS-3LT trinocular inverse microscope, Optika, Ponteranica, Italy) with 400× magnification.

### 2.4. Cell Viability

Cell viability was evaluated through trypan blue staining [20]. Briefly, amniocytes were mixed with 0.4% trypan blue, and under an optical microscope, we distinguished viable cells with clear cytoplasm from non-viable cells that incorporated the dye, with a blue cytoplasm. The number of viable cells was calculated as follows: Cell viability = (number of non-blue cells/number of total cells) × 100.

### 2.5. DNA Polymorphisms and Genomic Template Stability

The RAPD-PCR technique is a PCR that allows the electrophoretic polymorphic profiling of each sample by using a single short primer under low-annealing conditions. This technique allows verifying induced mutations in terms of the appearance and disappearance of polymorphic bands compared to untreated samples [21]. Genomic amniocyte DNA was isolated and purified from 200 µL cell suspensions using the High Pure PCR Template Preparation Kit (ROCHE Diagnostics^®^). The DNA amplification reaction was conducted using Primer 6 (5′-d[CCCGTCAGCA]-3′) according to Mottola and collaborators, 2019 [15]. Specifically, the amplification program comprised an initial step at 94 °C for 2 min, and then 1 min at 95 °C, 1 min at 36 °C and 2 min at 72 °C, for 45 cycles. A 1.5% agarose gel, stained with 1% ethidium bromide, was used for the electrophoresis of the amplification products. The RAPD-PCR profiles were used for GTS% calculation as follows: GTS = (1 − *a*/*n*) × 100, where *a* is the average number of polymorphic bands (the appearance of new bands and disappearance of bands) of each treated sample and *n* is the total band number in the negative control. The variations of these values were estimated as a percentage with respect to the control, set to 100% [22].

Binary coded characters (1, 0) were used for the molecular genetic investigation and elaborated using the Genesis software (Graz University of Technology Institute for Genomics and Bioinformatics 1.8.1).

### 2.6. DNA Fragmentation

To directly detect breaks in the amniocyte DNA in a simple and standardised manner [23], we used TUNEL tests (In Situ Cell Death Detection Kit, Fluorescein (ROCHE Diagnostics, Basel, Switzerland)) [15]. First, 10 µL of cell suspension, obtained after detachment with 1× trypsin and washing with 1× phosphate buffered saline (PBS), was gently smeared on slides previously cleaned with ethanol. Then, the slides were fixed in 4% paraformaldehyde for 1 h at room temperature (RT). After the fixing times, the slides were washed in 1 PBS and allowed to incubate in a permeabilizing solution (0.1% sodium citrate, 0.1% Triton X-100) for 30 min. The slides were washed in 1× PBS twice, and then, the water around the sample was allowed to dry. Next, 50 µL of the TUNEL reaction mixture (5 µL of terminal deoxy nucleotidyl transferase enzyme solution and 45 µL of Label Solution), previously prepared and left on ice, was added to each slide, which was then incubated in a humid chamber for 1 h at 37 °C in the dark. Then, the slides were washed three times in 1× PBS and stained in 4′, 6-diamidino-2-phenylindole (DAPI) for 5 min in the dark. Afterwards, 100 µL of 1,4 diazobicyclo (2,2,2) octane (DABCO) solution (20×) was added to each slide. Finally, the slides were observed under a fluorescent microscope, Nikon Eclipse E-600, equipped with BP 330–380 nm and LP 420 nm filters. About 350 cells per slide were analysed, determining the percentage of nuclei with fragmented DNA that incorporated the fluorescein, which appeared green, from the intact nuclei, which appeared blue (DFI = (number of green nuclei/number of total nuclei) × 100).

### 2.7. Statistical Analysis

The data are expressed as mean and standard deviation (SD). Differences in the percentages of cell viability, DNA fragmentation and genomic stability among the experimental groups were analysed by applying ANOVA (analysis of variance) tests with GraphPad Prism 6. Only results with *p*-value ≤ 0.05 (*p* ≤ 0.05) were considered statistically significant.

## 3. Results

### 3.1. Cell Morphology Analysis

The control amniocytes showed epithelioid and fibroblastoid-like phenotypes: cells with a usual elongated hexagonal shape with clear contours showing confluence were observed.

LA (300 µM) alone resulted in small cell morphology changes, while that in combination with 10 mM H_2_O_2_ induced changes in cell phenotype starting from short exposure times. In particular, there was the detachment of some cells from the culture system’s adhesion surface, with dark contours and cytoplasm.

The cells treated with LA (200 µM) presented clear contours and the classic elongated shape. The combination of 200 µM LA and 10 mM H_2_O_2_ showed some protective action of LA against H_2_O_2_, as the amniotic cells showed a regular morphology and phenotype.

**Figure 1:**
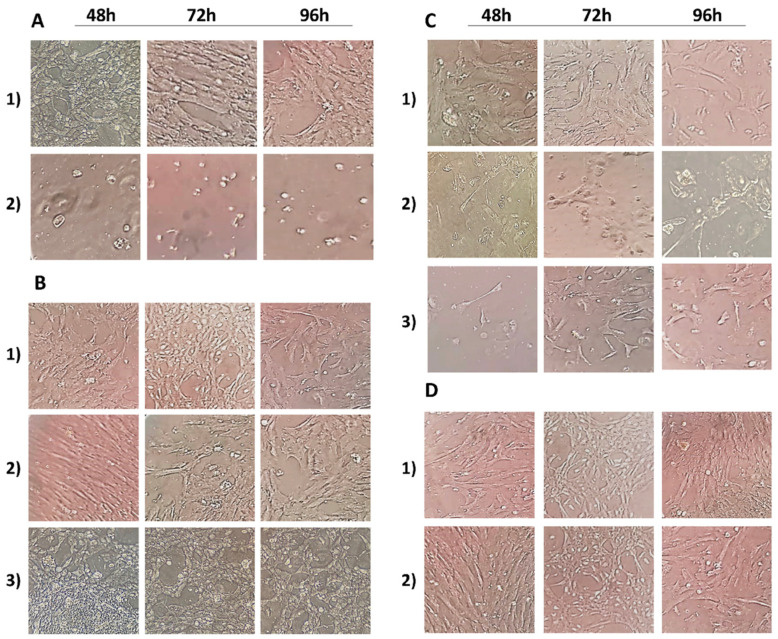
Amniotic cell morphology after different periods of exposure (48, 72 and 96 h) of all substances tested. (**A1**) Negative control; (**A2**) positive control (10 mM H_2_O_2_); (**B1**) 20 µM curcumin exposure; (**B2**) 40 µM curcumin exposure; (**B3**) 10 mM H_2_O_2_ + 40 µM curcumin co-exposure. (**C1**) 200 µM α-lipoic acid exposure; (**C2**) 300 µM α-lipoic acid exposure; (**C3**) 10 mM H_2_O_2_ + 200 µM α-lipoic acid co-exposure. (**D1**) 200 µM α-lipoic acid plus 40 µM curcumin co-treated amniotic cells; (**D2**) 200 µM α- lipoic acid plus 40 µM curcumin with 10 mM H_2_O_2_ co-treated amniotic cells.

Cur at 20 and 40 µM did not induce any morphological changes compared to the control cells at 48, 72 and 96 h. Treatment with 40 µM curcumin plus 10 mM H_2_O_2_ did not alter the cell morphology or phenotype at any of the exposure times tested, showing better cell phenotype protection than LA alone. Furthermore, 40 µM curcumin with 200 µM LA, including when both were combined with 10 mM H_2_O_2_ resulted in a regular fibroblastoid-like phenotype for the amniocytes for all the treatment times in contrast with H_2_O_2_ single treatment (Figure 1).

### 3.2. Cell Viability

No significant difference was observed in the percentage of viable amniotic cells after α-lipoic acid (200 and 300 µM) and curcumin (20 and 40 µM) exposure for all the times.

**Figure 2:**
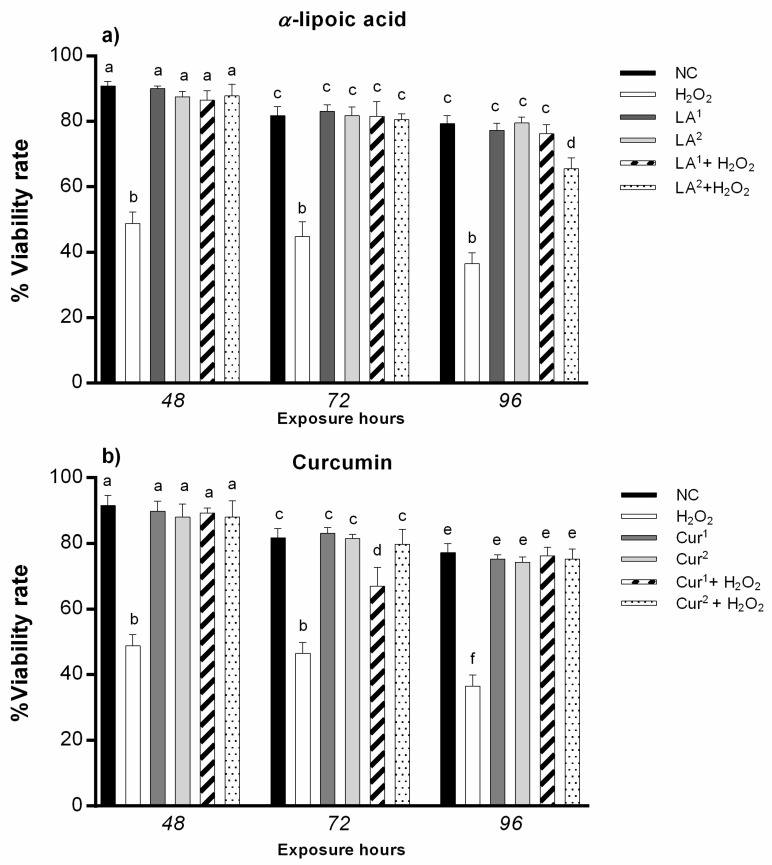
(**a**) Percentages of alive amniotic cells (ordinate) after different hours of exposure (abscissa) to H_2_O_2_, α-lipoic acid (LA) and H_2_O_2_ and LA (co-exposure). The black bars are negative control (NC); the white bars are positive control (10 mM H_2_O_2_); the dark grey bars are 200 µM α-lipoic acid (LA^1^); the light grey bars are 300 µM α-lipoic acid (LA^2^); the striped bars are amniotic cells co-treated with 10 mM H_2_O_2_ + 200 µM α-lipoic acid (LA^1^ + H_2_O_2_); the dotted bars are amniotic cells co-treated with 10 mM H_2_O_2_ + 300 µM α-lipoic acid (LA^2^ + H_2_O_2_). (**b**) Percentage of alive amniotic cells (ordinate) after different hours of exposure (abscissa) to H_2_O_2_, curcumin (Cur) and H_2_O_2_ and Cur combined. The black bars are negative control (NC); the white bars are positive control (10 mM H_2_O_2_); the dark grey bars are 20 µM curcumin (Cur^1^); the light grey bars are 40 µM curcumin (Cur^2^); the striped bars are amniotic cells co-treated with 10 mM H_2_O_2_ + 20 µM curcumin (Cur^1^ + H_2_O_2_); the dotted bars are amniotic cells co-treated with 10 mM H_2_O_2_ + 40 µM curcumin (Cur^2^ + H_2_O_2_). Number of viable cells was calculated as follows: Cell viability = (number of non-blue cells/number of total cells) × 100. Values with different letters differ with statistical significance (ANOVA)*. p* ≤ 0.05.

Co-exposure to 200 µM LA and 10 mM H_2_O_2_ did not induce statistically significant changes in viability, while 300 µM LA and 10 mM H_2_O_2_ co-exposure statistically significantly reduced amniotic cell viability after 96 h (Figure 2a).

The combination of 20 µM curcumin and 10 mM H_2_O_2_ induced a statistically significant reduction in viability after only 72 h, while 40 µM curcumin and 10 mM H_2_O_2_ co-treatment did not alter amniocyte viability (Figure 2b).

**Figure 3:**
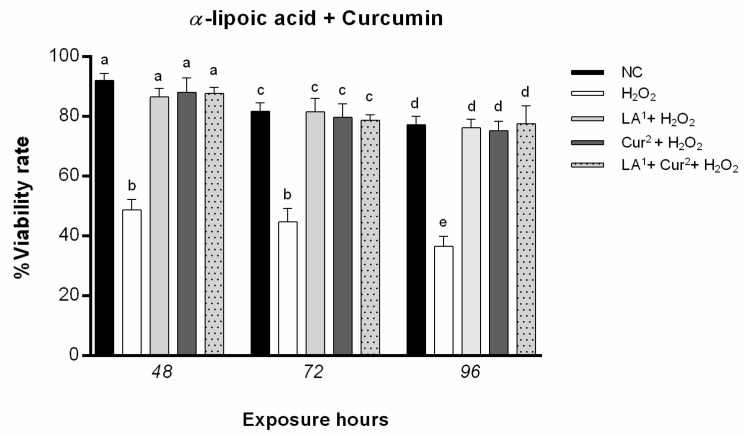
Percentages of alive amniotic cells (ordinate) after different hours of exposure (abscissa) to H_2_O_2_, H_2_O_2_ and LA (co-exposure), H_2_O_2_ and curcumin (Cur) (co-exposure) and LA plus Cur and H_2_O_2_. The black bars are negative control (NC); the white bars are positive control (10 mM H_2_O_2_); the light grey bars are 10 mM H_2_O_2_ + 200 µM α-lipoic acid (LA^1^ + H_2_O_2_); the dark grey bars are 10 mM H_2_O_2_ + 40 µM curcumin (Cur^2^ + H_2_O_2_); the grey dotted bars are amniotic cells co-treated with 200 µM α-lipoic acid plus 40 µM curcumin with 10 mM H_2_O_2_ (LA^1^ + Cur^2^ + H_2_O_2_). Number of viable cells was calculated as follows: Cell viability = (number of non-blue cells/number of total cells) × 100. Values with different letters differ with statistical significance (ANOVA). *p* ≤ 0.05.

Co-exposure to 200 µM α-lipoic acid and 40 µM curcumin for 48, 72 and 96 h did not statistically significantly reduce amniotic cell viability, nor did co-treatment with 200 µM α-lipoic acid plus 40 µM α-curcumin with 10 mM H_2_O_2_ reduce cell viability (Figure 3).

### 3.3. DNA Polymorphic Profiles

**Figure 4:**
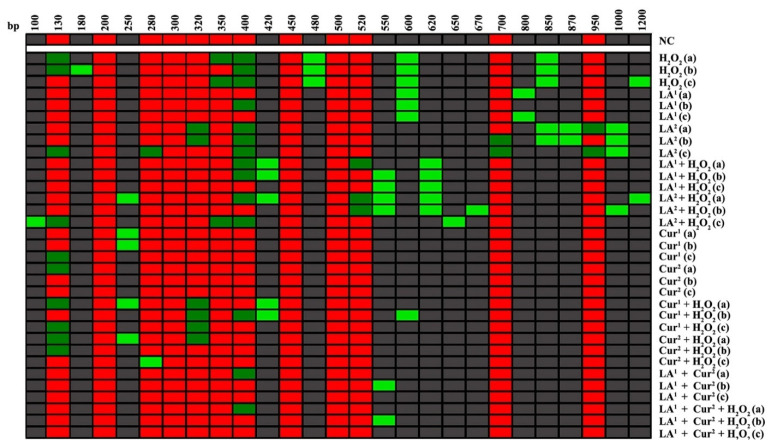
Molecular sizes (bp), as determined by Genesis software, of bands appearing and disappearing after amplification with primer 6 for DNA of human amniocytes exposed to different substances for (a) 48 h, (b) 72 h or (c) 96 h. NC: negative control; H_2_O_2_: 10 mM hydrogen peroxide; LA^1^: 200 µM α-lipoic acid; LA^2^: 300 µM α-lipoic acid; Cur^1^: 20 µM curcumin; Cur^2^: 40 µM curcumin. Light green: new bands that appeared; dark green: disappeared bands; red: bands not changed with respect to the control.

The RAPD-PCR analysis showed a variation of the polymorphic profiles of the DNA of amniocytes exposed to LA and Cur alone and in combination with 10 mM H_2_O_2_. LA at 200 µM induced a variation of only 2 bands for all the exposure times, unlike 300 µM LA, which induced a deep change in polymorphic bands equal to the positive control (10 mM H_2_O_2_). After 24 and 72 h, 200 µM LA with 10 mM H_2_O_2_ produced variation in four bands, while 96 h of exposure to 200 µM LA and 10 mM H_2_O_2_ generated only two new bands. The electrophoretic patterns for 300 µM LA with 10 mM H_2_O_2_ co-exposure showed a pronounced band alteration compared to non-treated amniocytes. The exposure to curcumin caused slight differences in total band numbers; even 40 µM Cur did not provoke any band disappearance and/or appearance after 72 and 96 h. Co-treatment with 20 µM Cur and 10 mM H_2_O_2_ produced variation in four bands after 24 and 72 h, while only the disappearance of two bands after 96 h, with respect to the control, was observed. Co-exposure to 40 µM Cur and 10 mM H_2_O_2_ for 24 h provoked a change in three bands; by contrast, 72 and 96 h induced only one band alteration. The RAPD-PCR polymorphic patterns related to 200 µM LA plus 40 µM Cur co-exposure were the same as those obtained for 200 µM LA and 40 µM Cur plus 10 mM H_2_O_2_, reaching the negative control polymorphic profiles after 96 h of co-exposure (Figure 4).

### 3.4. Genomic Template Stability

**Figure 5:**
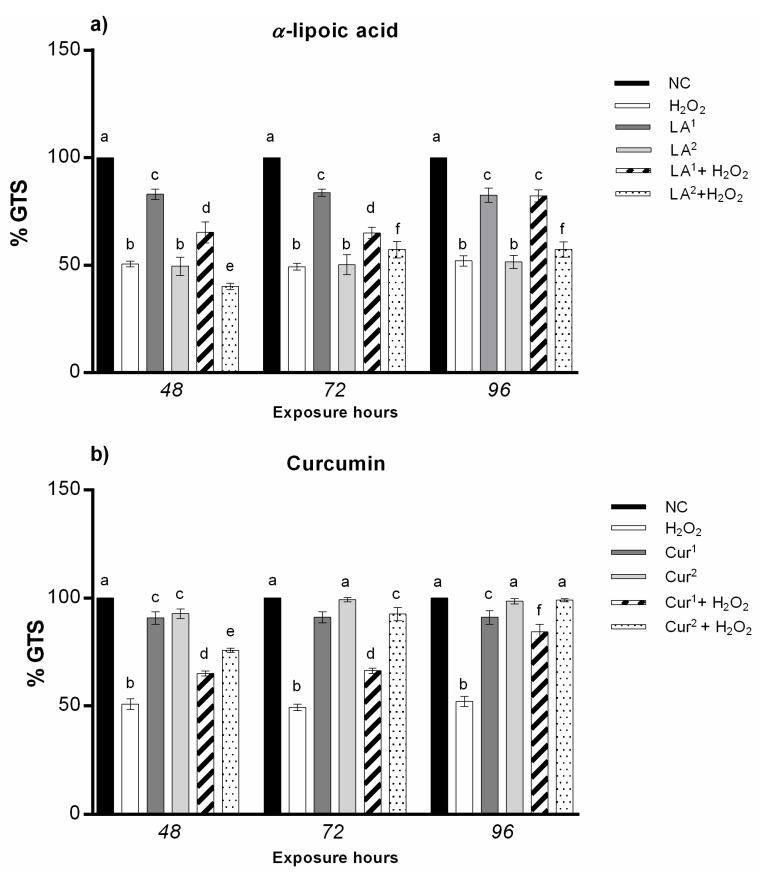
(**a**) Changes in the percentage of genome template stability (ordinate) in amniotic cells after different hours of exposure (abscissa) to H_2_O_2_, α-lipoic acid (LA) and H_2_O_2_ and LA combined. The black bars are negative control (NC); the white bars are positive control (10 mM H_2_O_2_); the dark grey bars are 200 µM α-lipoic acid (LA^1^); the light grey bars are 300 µM α-lipoic acid (LA^2^); the striped bars are 10 mM H_2_O_2_ + 200 µM co-treatment (LA^1^ + H_2_O_2_); the dotted bars are 10 mM H_2_O_2_ + 300 µM α-lipoic acid co-treatment (LA^2^ + H_2_O_2_). (**b**) Changes in percentage of genome template stability (ordinate) in amniotic cells after different hours of exposure (abscissa) to H_2_O_2_, curcumin (Cur) and H_2_O_2_ and Cur combined. The black bars are negative control (NC); the white bars are positive control (10 mM H_2_O_2_); the dark grey bars are 20 µM curcumin (Cur^1^); the light grey bars are 40 µM curcumin (Cur^2^); the striped bars are 10 mM H_2_O_2_ + 20 µM curcumin co-treatment (Cur^1^ + H_2_O_2_); the dotted bars are 10 mM H_2_O_2_ + 40 µM curcumin co-treatment (Cur^2^ + H_2_O_2_). GTS% was calculated as follows: GTS = (1 − *a*/*n*) × 100, where *a* is the average number of polymorphic bands (the appearance of new bands and disappearance of bands) of each treated sample and *n* is the total band number in the negative control. The variations of these values were estimated as percentages with respect to the control, set to 100%. Values with different letters differ with statistical significance (ANOVA). *p* ≤ 0.05.

The analysis of the RAPD DNA polymorphic profiles allowed us to calculate the amniocyte genome stability percentage (GTS%). α-lipoic acid at 200 µM induced a statistically significant reduction in DNA stability after only 48 h of exposure, while 300 µM α-lipoic acid provoked genome instability at all the exposure times (48, 72 and 96 h). The results indicate that amniocytes’ genomic stability only increases with α-lipoic acid (200 µM) and H_2_O_2_ co-treatment with respect to H_2_O_2_ alone, until it reaches values not statistically significant after long exposure hours (96 h) (Figure 5a).

The exposure to 20 and 40 µM curcumin did not reduce the genome stability in amniotic cells in vitro for any of the times. The combination of 20 µM curcumin with H_2_O_2_ preserved DNA stability with respect to H_2_O_2_ alone, with values not statistically significant after the maximum exposure time (96 h); the co-treatment with 40 µM curcumin plus H_2_O_2_ enhanced the GTS% in amniotic cells starting from 72 h, which reached negative control values at 96 h (Figure 5b).

**Figure 6:**
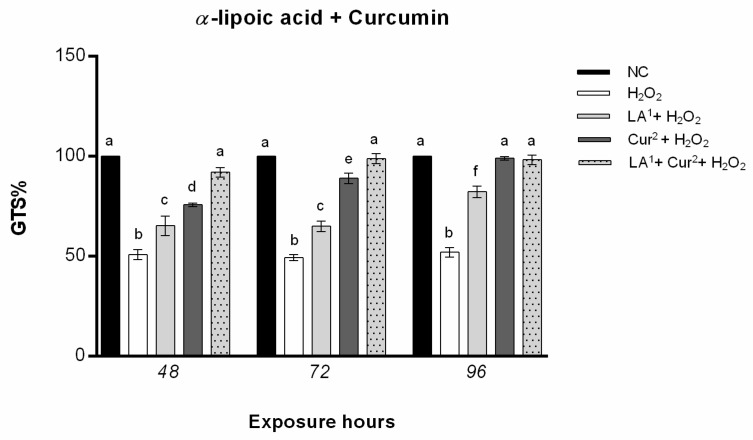
Change in percentage of genome template stability (ordinate) in amniotic cells after different hours of exposure (abscissa) to H_2_O_2_, H_2_O_2_ and LA (co-exposure), H_2_O_2_ and curcumin (Cur) (co-exposure) and LA plus Cur and H_2_O_2_. The black bars are negative control (NC); the white bars are positive control (10 mM H_2_O_2_); the light grey bars are 10 mM H_2_O_2_ + 200 µM α-lipoic acid (LA^1^ + H_2_O_2_); the dark grey bars are 10 mM H_2_O_2_ + 40 µM curcumin (Cur^2^ + H_2_O_2_); the grey dotted bars are 200 µM α-lipoic acid plus 40 µM curcumin with 10 mM H_2_O_2_ co-treatment (LA^1^ + Cur^2^ + H_2_O_2_). GTS% was calculated as follows: GTS = (1 − *a*/*n*) × 100, where *a* is the average number of polymorphic bands (the appearance of new bands and disappearance of bands) of each treated sample and *n* is the total band number in the negative control. The variations of these values were estimated as percentages with respect to the control, set to 100%. Values with different letters differ with statistical significance (ANOVA). *p* ≤ 0.05.

The combination of 200 µM α-lipoic acid, 40 µM curcumin and H_2_O_2_ (10 mM) increased amniocytes’ DNA stability until it almost reached the negative control level for all the exposure hours (Figure 6).

### 3.5. DNA Fragmentation

The results from the TUNEL test showed that exposure to 200 µM LA did not result in statistically significant DNA damage. By contrast, 300 µM LA induced a statistically significant increase in the amniotic cells’ DNA fragmentation index (DFI) after all the exposure hours alone and in combination with 10 mM H_2_O_2_.

**Figure 7:**
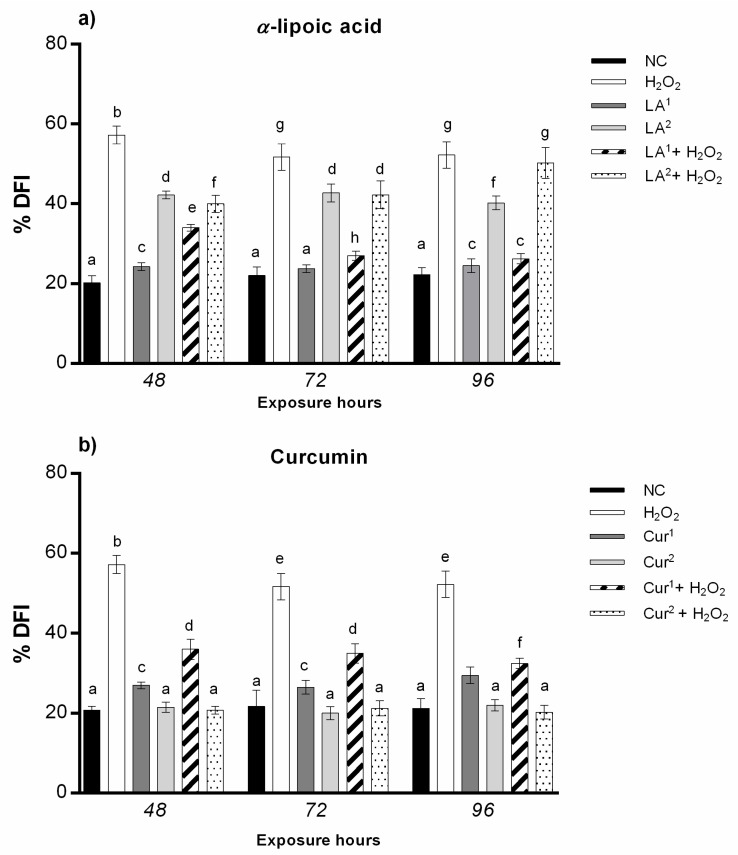
(**a**) Percentage of DNA fragmentation (ordinate) in amniotic cells after different hours of exposure (abscissa) to H_2_O_2_, α-lipoic acid (LA) and H_2_O_2_ and LA combined. The black bars are negative control (NC); the white bars are positive control (10 mM H_2_O_2_); the dark grey bars are 200 µM α-lipoic acid (LA^1^); the light grey bars are 300 µM α-lipoic acid (LA^2^); the striped bars are 10 mM H_2_O_2_ + 200 µM α-lipoic acid co-treatment (LA^1^ + H_2_O_2_); the dotted bars are 10 mM H_2_O_2_ + 300 µM α-lipoic acid co-treatment (LA^2^ + H_2_O_2_). (**b**) Percentage of DNA fragmentation (ordinate) in amniotic cells after different hours of exposure (abscissa) to H_2_O_2_, curcumin (Cur) and H_2_O_2_ and Cur combined. The black bars are negative control (NC); the white bars are positive control (10 mM H_2_O_2_); the dark grey bars are 20 µM curcumin (Cur^1^); the light grey bars are 40 µM curcumin (Cur^2^); the striped bars are 10 mM H_2_O_2_ + 20 µM curcumin co-treatment (Cur^1^ + H_2_O_2_); the dotted bars are 10 mM H_2_O_2_ + 40 µM curcumin co-treatment (Cur^2^ + H_2_O_2_). DNA fragmentation index was calculated as follows: DFI = (number of green nuclei/number of total nuclei) × 100). Values with different letters differ with statistical significance (ANOVA). *p* ≤ 0.05.

Co-exposure to 200 µM LA and 10 mM H_2_O_2_ caused DNA fragmentation in amniocytes only after 48 h, while no statistically significant DNA damage was observed from 72 to 96 h (Figure 7a).

Cur treatment at both concentration (20 and 40 µM) did not induce amniocyte DNA fragmentation for any of the exposure hours. The combination of 20 µM curcumin and 10 mM H_2_O_2_ caused a reduction in DNA fragmentation with respect to H_2_O_2_ alone with values not statistically significant after a prolonged exposure time (96 h), whereas 40 µM curcumin and 10 mM H_2_O_2_ co-exposure reduced the DFI in amniotic cells from 48 h (Figure 7b).

**Figure 8:**
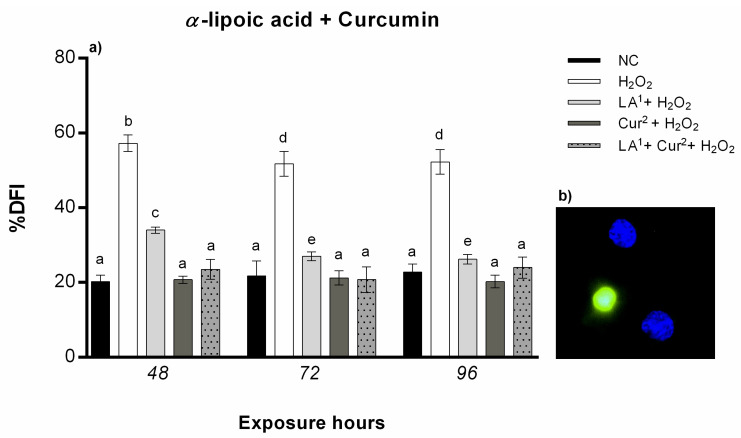
(**a**) Percentage of DNA fragmentation (ordinate) in amniotic cells after different hours of exposure (abscissa) to H_2_O_2_, H_2_O_2_ and LA (co-exposure), H_2_O_2_ and curcumin (Cur) (co-exposure) and LA plus Cur and H_2_O_2_. The black bars are negative control (NC); the white bars are positive control (10 mM H_2_O_2_); the light grey bars are 10 mM H_2_O_2_ + 200 µM α-lipoic acid (LA^1^ + H_2_O_2_); the dark grey bars are 10 mM H_2_O_2_ + 40 µM curcumin (Cur^2^ + H_2_O_2_); the grey dotted bars are 200 µM α-lipoic acid plus 40 µM curcumin with 10 mM H_2_O_2_ co-treatment (LA^1^ + Cur^2^ + H_2_O_2_). DNA fragmentation index was calculated as follows: DFI = (number of green nuclei/number of total nuclei) × 100). Values with different letters differ with statistical significance (ANOVA). *p* ≤ 0.05. (**b**) Amniotic cells with fragmented DNA (green) and intact DNA (blue), after H_2_O_2_ treatment, detected with Nikon E600 fluorescence microscope.

The combination of 200 µM α-lipoic acid and 40 µM curcumin with 10 mM H_2_O_2_ statistically significantly reduced the DNA fragmentation in amniocytes with respect to H_2_O_2_ alone for all the exposure hours (Figure 8).

## 4. Discussion

The ability of free radicals to induce DNA damage, as well as their link to chronic-degenerative pathologies, has greatly expanded the research on natural or synthetic molecules capable of limiting or preventing the potential damage caused by ROS.

Antioxidants improve the body’s immune defences and reduce the risk of disease, preventing ROS damage to biological macromolecules [24,25].

Given the serious consequences induced by ROS in cells, tissues and organs, living organisms have developed a complex antioxidant defence system to preserve the stability of their cellular structures. Despite its remarkable efficiency, the endogenous antioxidant system is not sufficient to keep the concentrations of free radicals low; therefore, humans need to introduce different types of antioxidants into their diets. Exogenous antioxidants are increasingly used to combat OS. OS is the undesirable effect of the biochemical balance between oxidizing species and antioxidant molecules being disrupted, which can influence the onset and/or courses of many inflammatory or degenerative diseases, including those occurring in natural conditions such as pregnancy [26].

The monitoring of certain substances within the amniotic fluid has highlighted the importance of vitamin intake during pregnancy; in particular, low levels of folic acid and vitamin B12 were found in the amniotic fluid of foetuses with neural tube malformations [27]. The detection of these substances in pregnancy has been shown to be useful in some diseases linked to oxidative stress, such as celiac disease [28]. This pathology induces malabsorption and deficiencies of vitamins (folic acid and vitamins B12, K and B6) and minerals important for prenatal development, leading to serious foetal and neonatal consequences [29].

The opportunity to monitor the antioxidant and vitamin “reserves” of the foetus, through the amniotic fluid, could provide the opportunity to intervene, before delivery, by restoring nutritional levels and thus promoting correct foetal development.

Numerous studies have been undertaken to identify new plant resources with low side effects and strong antioxidant properties that protect against cell damage.

Our study focused on LA and Cur, two amphipathic antioxidant substances capable of acting in different areas of the organism, as they can cross biological membranes, including the blood–brain barrier [30,31,32].

Our results showed the ability of 200 µM LA to counteract H_2_O_2_′s genotoxic effect on human amniocyte cultures in vitro at the molecular level and in terms of DNA breaks. About 200 µM LA showed protection against cell death, as it was able to reduce the percentage of cells with fragmented DNA at prolonged exposure times, suggesting time-dependent action and cell death protection. At the same times, LA significantly increased genomic stability compared to the oxidizing agent alone (H_2_O_2_), and in this case, the protective action was also time-dependent, with greater antigenotoxic power for prolonged times of exposure. The ability of LA to neutralise genotoxicity is in agreement with an in vitro study conducted on human placental trophoblasts that demonstrated LA’s ability to counteract cellular apoptosis [33], which occurs in the placenta in hypoxic conditions [34]. By contrast, the data showed that 300 µM LA provoked a statistically significant increase in amniotic cells with fragmentated DNA and genome instability after all the exposure hours. These results show that antioxidants can induce genotoxicity when applied beyond certain concentrations, probably because they can reduce ROS below physiological concentrations, preventing the latter from carrying out their biological functions. However, it is necessary to determine the appropriate dose(s) to be used to maximise the beneficial antioxidant and detoxifying effects and to minimise any pro-oxidant toxic effects. Therefore, future research will be aimed at evaluating the effects of LA concentrations less than 300 µM.

Cur also showed an ability to counteract the H_2_O_2_-induced oxidative damage, particularly at the highest concentration tested (40 µM). Literature data have already shown the protective effects of curcumin against damage induced by mutagenic pollutants such as benzo(a)pyrene and other genotoxic agents, protecting against DNA fragmentation by suppressing ROS production and increasing glutathione peroxidase 4 (GPX4) gene expression [35], which is known to protect cells from DNA-damaging oxidative stress caused by cell membrane peroxidation [36].

Our results confirm the anticytotoxic and antigenotoxic potential of curcumin, as cell morphology and phenotype were preserved for all the treatment times. Furthermore, a statistically significant reduction in the DFI produced by 40 µM Cur with respect to exposure to H_2_O_2_ alone was observed. In particular, the results show significant reductions in the percentage of cells with fragmentated DNA and induced mutations for the intermediate and maximum exposure times tested, indicating a mechanism of action that requires prolonged times.

Regarding H_2_O_2_ genotoxicity, the results of this research are in agreement with the literature, as H_2_O_2_ is responsible for DNA damage [37,38]; more specifically, the results show the susceptibility of foetal cells to ROS’ negative effects, both triggering apoptosis and reducing genomic stability, sometimes by over 50%. To protect cells and, consequently, DNA from this evident damage, in some cases, the action of a single antioxidant molecule is not enough. In fact, some data report that antioxidants work in a synergistic manner when administered simultaneously [39,40,41]. It is known that vitamin E requires vitamin C supplementation to generate the maximum antioxidant effect [42]. In the same way, an interaction between astaxanthin and vitamin E produces a synergistic antioxidant effect [43].

From our results, the association of 200 µM LA and 40 µM Cur showed a high anticytotoxic and antigenotoxic effect, although only at the maximum exposure times, in fact, while the viability and cell morphology, were preserved for all the times, the percentage of cells with fragmented DNA and the genome stability reached values similar to those for the negative control after 96 h of combined treatment (LA plus Cur) with H_2_O_2_, greatly reducing the genotoxic activity of the latter. However, 40 µM Cur seems to have alone the ability to counteract H_2_O_2_ genotoxicity, indeed restoring the negative control status both in DFI and GTS%, especially after longer time of exposure. So, future studies through combination index will be needed to analyse the potential synergy between LA and Cur.

The association of Cur and LA, being able to defend cells and DNA against ROS-induced mutations in terms of the GTS%, DFI and amniocyte viability in vitro, could represent an innovative approach to protecting foetal DNA from OS during pregnancy. This evidence arouses interest in investigating the potential combined effects of the two antioxidants in protecting DNA against gene mutations, through involvement in the mechanisms of the detoxification of free radicals, in the prenatal phase. These results could provide a valid starting point for the therapeutic prevention of prenatal OS and pathologies associated with genetic disorders.

Although our study has highlighted the beneficial effects of these two molecules against oxidative damage, further studies are still needed to investigate the effects of the molecules in question on DNA. In fact, this study showed a greater antigenotoxic ability of 40 µM Cur compared to 20 µM and compared to LA alone. However, especially in view of a real future therapy, it should be noted that exposure to individual molecules induced variations, even though minimal, compared to controls, in terms of DNA adducts, as highlighted with the RAPD-PCR technique. Therefore, further tests with the use of different concentrations of these antioxidant molecules will be fundamental, as well as investigation with lower concentration of antioxidants are necessary to get antigenotoxic effects more easily reached in vivo.

It is known that even an antioxidant compound can have deleterious effects if administered in excessive doses, and in fact, our results indicate that the supplementation of 300 µM LA provokes conspicuous DNA damage in amniotic cells similar to that induced by H_2_O_2_. A documented example of this phenomenon is provided by retinoic acid, a derivative of vitamin A, which, despite participating in the formation of the anteroposterior axis of the mammalian embryo and in the formation of limbs, if present in high concentrations, can have serious negative repercussions for embryonic development [44].

At the same time, experiments with a mouse model revealed that the consumption of 40 μM curcumin resulted in reduced maturation of oocytes and alterations in embryonic development [45].

Furthermore, even though in vivo studies for human risk assessment are limited, it must be considered that polyphenols can inhibit oestrogen metabolism, which can even lead to an increase in the proliferation of breast cancer cells [46].

Therefore, it is not easy to translate the effects obtained in vitro into potential health benefits. It must be considered that the bioavailability of food flavonoids is influenced by several factors including metabolism, absorption, water solubility, cell membrane permeability and their ability to cross the blood–brain barrier [47].

Therefore, further studies in in vivo models will be needed to determine the appropriate doses of LA and Cur to use as therapy, testing a wide range of concentrations to ensure their bioavailability and also to evaluate their actual ability to cross the placental barrier. It is known that LA has rapid uptake and low bioavailability, and the metabolism is primarily hepatic [48,49]; additionally, Cur exhibits poor bioavailability due to poor absorption, rapid metabolism and rapid systemic elimination [50].

This evidence underlines the need for more investigations into the antigenotoxic or genotoxic effects of LA and Cur metabolites, as well as clinical trials to assess the long-term safety of LA and Cur, before such antioxidant treatment is extended to pregnant women.

## 5. Conclusions

Natural products offer an incalculable variety of chemical compounds, and it is likely that phytochemicals will acquire more interest as important therapeutic factors for various degenerative diseases. The therapeutic applications of substances of natural origin are forming the basis of modern medical science and the starting point for the birth of new drugs. Diet is closely linked to both the incidence and prevention of many types of diseases associated with OS. Appropriate eating behaviour as well as the integration of antioxidant supplements during pregnancy can reduce the risk of pathologies related to oxidative stress in the unborn child. Our results show a protective action of LA at 200 µM against oxidative DNA damage. Similarly, Cur is able alone to counteract H_2_O_2_ genotoxicity, resulting in an increase in genomic stability and reduction in the DNA fragmentation index in human amniotic cells in vitro. Moreover, the combination of 40 µM cur and 200 µM LA has a high antigenotoxic effect, with a strong increase in genomic stability, reaching values close to those for the negative control and a significant decrease in the DFI. It must be considered that this evidence for the effects of LA and Cur is limited to an in vitro system, so further studies on in vivo models may be necessary to realise the use of these antioxidants during pregnancy. Therefore, the extraordinary antioxidant and protective actions of LA and Cur in vitro suggest their possible use for beneficial intervention in the context of oxidative imbalances resulting from endogenous or external insults during pregnancy, exerting a protective action against permanent mutations in the foetus induced by oxygen free radicals.

## Data Availability

Data is contained within the article.

## References

[1] Cooke M.S., Evans M.D., Dizdaroglu M., Lunec J. (2003). Oxidative DNA damage: Mechanisms, mutation, and disease. FASEB J..

[2] Poulsen Henrik E. (2005). Oxidative DNA modifications. Exp. Toxicol. Pathol..

[3] Hu X.Q., Zhang L. (2021). Hypoxia and mitochondrial dysfunction in pregnancy complications. Antioxidants.

[4] Hung T.H., Burton G.J. (2006). Hypoxia and reoxygenation: A possible mechanism for placental oxidative stress in preeclampsia. Taiwan J. Obstet. Gynecol..

[5] Cuffe J.S., Xu Z.C., Perkins A.V. (2017). Biomarkers of oxidative stress in pregnancy complications. Biomark. Med..

[6] Ramkumar M. (2014). Oxidative stress damage as a detrimental factor in preterm birth pathology. Front. Immunol..

[7] Cetin I., Berti C., Calabrese S. (2010). Role of micronutrients in the periconceptional period. Hum. Reprod. Update.

[8] Conde-Agudelo A., Romero R., Kusanovic J.P., Hassan S.S. (2011). Supplementation with vitamins C and E during pregnancy for the prevention of preeclampsia and other adverse maternal and perinatal outcomes: A systematic review and metaanalysis. Am. J. Obstet. Gynecol..

[9] Formoso G., Baldassarre M.P.A., Ginestra F., Carlucci M.A., Bucci I., Consoli A. (2019). Inositol and antioxidant supplementation: Safety and efficacy in pregnancy. Diabetes Metab. Res. Rev..

[10] Costantino D., Guaraldi C., Costantino M., Bounous V.E. (2015). Acido alfa-lipoico e omega-3 nel trattamento del dolore nel postpartum (Use of alpha-lipoic acid and omega-3 in postpartum pain treatment). Minerva Ginecol..

[11] Soleimani V., Sahebkar A., Hosseinzadeh H. (2018). Turmeric (Curcuma longa) and its major constituent (curcumin) as nontoxic and safe substances: Review. Phytother. Res..

[12] Filardi T., Varì R., Ferretti E., Zicari A., Morano S., Santangelo C. (2020). Curcumin: Could this compound be useful in pregnancy and pregnancy-related complications?. Nutrients.

[13] Zhou J., Miao H., Li X., Hu Y., Sun H., Hou Y. (2017). Curcumin inhibits placental inflammation to ameliorate LPS-induced adverse pregnancy outcomes in mice via upregulation of phosphorylated Akt. Inflamm. Res..

[14] Hoseini S.M., Kalantar S.M., Bahrami A.R., Matin M.M. (2020). Human amniocytes: A comprehensive study on morphology, frequency and growth properties of subpopulations from a single clone to the senescence. Cell Tiss. Biol..

[15] Mottola F., Iovine C., Santonastaso M., Romeo M.L., Pacifico S., Cobellis L., Rocco L. (2019). NPs-TiO_2_ and Lincomycin coexposure induces DNA damage in cultured human amniotic cells. Nanomaterials.

[16] Lin X., Bai D., Wei Z., Zhang Y., Huang Y., Deng H., Huang X. (2019). Curcumin attenuates oxidative stress in RAW264.7 cells by increasing the activity of antioxidant enzymes and activating the Nrf2-Keap1 pathway. PLoS ONE.

[17] Unal F., Taner G., Yuzbasioglu D., Yilmaz S. (2013). Antigenotoxic effect of lipoic acid against mitomycin-C in human lymphocyte cultures. Cytotechnology.

[18] Maddux B.A., See W., Lawrence J.C.J., Goldfine A.L., Goldfine I.D., Evans J.L. (2001). Protection against oxidative stress-induced insulin resistance in rat L6 muscle cells by mircomolar concentrations of alpha-lipoic acid. Diabetes.

[19] Miki T., Lehmann T., Cai H., Stolz D.B., Strom S.C. (2005). Stem cell characteristics of amniotic epithelial cells. Stem Cells.

[20] Strober W. (2001). Trypan blue exclusion test of cell viability. Curr. Protoc. Immunol..

[21] Liu W., Yang Y.S., Zhou Q., Xie L., Li P., Sun T. (2007). Impact assessment of cadmium contamination on rice (*Oryza sativa* L.) seedlings at molecular and population levels using multiple biomarkers. Chemosphere.

[22] Santonastaso M., Mottola F., Iovine C., Cesaroni F., Colacurci N., Rocco L. (2020). In Vitro effects of Titanium Dioxide nanoparticles (TiO_2_NPs) on Cadmium Chloride (CdCl_2_) Genotoxicity in human sperm cells. Nanomaterials.

[23] Sharma R., Iovine C., Agarwal A., Henkel R. (2021). TUNEL assay-standardized method for testing sperm DNA fragmentation. Andrologia.

[24] Mottola F., Scudiero N., Iovine C., Santonastaso M., Rocco L. (2020). Protective activity of ellagic acid in counteract oxidative stress damage in zebrafish embryonic development. Ecotoxicol. Environ. Saf..

[25] Iovine C., Mottola F., Santonastaso M., Finelli R., Agarwal A., Rocco L. (2021). In vitro ameliorative effects of ellagic acid on vitality, motility and DNA quality in human spermatozoa. Mol. Reprod. Dev..

[26] Markesbery W.R. (1997). Oxidative stress hypothesis in Alzheimer’s disease. Free Radic. Biol. Med..

[27] Steen M.T., Boddie A.M., Fisher A.J., Macmahon W., Saxe D., Sullivan K.M., Dembure P.P., Elsas L.J. (1998). Neural-tube defects are associated with low concentration of cobalamine (vitamin B12) in amniotic fluid. Prenat. Diagn..

[28] Odetti P., Valentini S., Aragno I., Garibaldi S., Pronzato M.A., Rolandi E., Barreca T. (1998). Oxidative stress in subjects affected by celiac disease. Free Radic. Res..

[29] Dickey W., Stewart F., Nelson J., McBreen G., McMillan S.A., Porter K.G. (1996). Screening for coeliac disease as a possible maternal risk factor for neural tube defect. Clin. Genet..

[30] Cotman C.W., Head E., Muggenburg B.A., Zicker S., Milgram N.W. (2002). Brain aging in the canine: A diet enriched in antioxidants reduces cognitive dysfunction. Neurobiol. Aging.

[31] Toklu H.Z., Hakan T., Celik H., Biber N., Erzik C., Ogunc A.V., Akakin D., Cikler E., Cetinel S., Ersahin M. (2010). Neuroprotective effects of alpha-lipoic acid in experimental spinal cord injury in rats. J. Spinal Cord Med..

[32] Liczbiński P., Michałowicz J., Bukowska B. (2020). Molecular mechanism of curcumin action in signaling pathways: Review of the latest research. Phytother. Res..

[33] Wu H.Y., Lin C.Y., Chen T.C., Pan S.T., Yuan C.J. (2011). Mammalian Ste20-like protein kinase 3 plays a role in hypoxia-induced apoptosis of trophoblast cell line 3A-sub-E. Int. J. Biochem. Cell Biol..

[34] Hung T.H., Skepper J.N., Burton G.J. (2001). In vitro ischemia-reperfusion injury in term human placenta as a model for oxidative stress in pathological pregnancies. Am. J. Pathol..

[35] Santonastaso M., Mottola F., Iovine C., Colacurci N., Rocco L. (2021). Protective effects of Curcumin on the outcome of Cryopreservation in human sperm. Reprod. Sci..

[36] Brigelius-Flohé R., Maiorino M. (2013). Glutathione peroxidases. Biochim. Biophys. Acta.

[37] Marinho H.S., Real C., Cyrne L., Soares H., Antunes F. (2014). Hydrogen peroxide sensing, signaling and regulation of transcription factors. Redox Biol..

[38] Rosen J.E., Prahalad A.K., Williams G.M. (1996). 8-Oxodeoxyguanosine formation in the DNA of cultured cells after exposure to H_2_O_2_ alone or with UVB or UVA irradiation. Photochem. Photobiol..

[39] Trombino S., Serini S., Di Nicuolo F., Celleno L., Andò S., Picci N., Calviello G., Palozza P. (2004). Antioxidant effect of ferulic acid in isolated membranes and intact cells: Synergistic interactions with alpha-tocopherol, beta-carotene, and ascorbic acid. J. Agric. Food Chem..

[40] Torricelli P., Ricci P., Provenzano B., Lentini A., Tabolacci C. (2011). Synergic effect of α-tocopherol and naringenin in transglutaminase-induced differentiation of human prostate cancer cells. Amino Acids..

[41] Koekkoek W.A., van Zanten A.R. (2016). Antioxidant vitamins and trace elements in critical illness. Nutr. Clin. Pract..

[42] Robinson I., de Serna D.G., Gutierrez A., Schade D.S. (2006). Vitamin E in humans: An explanation of clinical trial failure. Endocr. Pract..

[43] Kogure K. (2019). Novel antioxidative activity of Astaxanthin and its synergistic effect with vitamin E. J. Nutr. Sci. Vitaminol..

[44] Lammer E.J., Chen D.T., Hoar R.M., Agnish N.D., Benke P.J., Braun J.T., Curry C.J., Fernhoff P.M., Grix A.W.J., Lott I.T. (1985). Retinoic acid embryopathy. N. Engl. J. Med..

[45] Chen C.C., Chan W.H. (2012). Injurious effects of curcumin on maturation of mouse oocytes, fertilization and fetal development via apoptosis. Int. J. Mol. Sci..

[46] Poschner S., Maier-Salamon A., Thalhammer T., Jäger W. (2019). Resveratrol and other dietary polyphenols are inhibitors of estrogen metabolism in human breast cancer cells. J. Steroid Biochem. Mol. Biol..

[47] Squillaro T., Schettino C., Sampaolo S., Galderisi U., Di Iorio G., Giordano A., Melone M.A.B. (2018). Adult-onset brain tumors and neurodegeneration: Are polyphenols protective?. J. Cell Physiol..

[48] Teichert J., Hermann R., Ruus P., Preiss R. (2003). Plasma kinetics, metabolism, and urinary excretion of alpha-lipoic acid following oral administration in healthy volunteers. J. Clin. Pharmacol..

[49] Shay K.P., Moreau R.F., Smith E.J., Smith A.R., Hagen T.M. (2009). Alpha-lipoic acid as a dietary supplement: Molecular mechanisms and therapeutic potential. Biochim. Biophys. Acta.

[50] Anand P., Kunnumakkara A.B., Newman R.A., Aggarwal B.B. (2007). Bioavailability of curcumin: Problems and promises. Mol. Pharm..

